# Characterization of pathways involved in colorectal cancer using real-time RT-PCR gene expression data 

**Published:** 2021

**Authors:** Samira Shabani, Nasibeh Khayer, Jamshid Motalebzadeh, Tayebeh Majidi zadeh, Frouzandeh Mahjoubi

**Affiliations:** 1 *Department of Clinical Genetic, National Institute of Genetic Engineering and Biotechnology (NIGEB), Tehran, Iran*; 2 *Skull Base Research Center, The Five Senses Health Institute, Iran University of Medical Sciences, Tehran, Iran *

**Keywords:** Colorectal cancer, Computational analysis, Real-time RT-PCR, Gene set enrichment analysis (GSEA), Gene regulatory network (GRN)

## Abstract

**Aim:**

Efforts to explore biomarkers and biological pathways involved in the disease are needed to improve colorectal cancer (CRC) diagnosis and alternative treatments

**Background:**

The fourth common malignancy in the world is colorectal cancer. The over-all burden is predicted to rise by 2030.

**Methods:**

In the current study, nine genes were selected. Previously, a panel of genes by Agendia, a classifier of robust gene expression (ColoPrint), was determined to significantly improve the prognostic accuracy of pathologic factors in stage II and III colorectal cancer patients. Five genes, including *Ppara, Mctp1, Pyroxd1, Il2r*, and *Cyfip2,* from this panel and four other genes which were not in this panel but were cited abundantly in the literature were selected. Then, expression levels of the selected genes in CRC tissue were compared with levels in adjacent normal tissue. To identify the pathways involved in CRC, gene set enrichment analysis was subsequently performed. Furthermore, to illustrate the relationship between genes in this disease, the cross-shaped co-expression pattern and gene regulatory network were determined using computational methods.

**Results:**

This research found that the pairs of genes: {*IL2R, CYFIP2*}, {*FOXM1*, *PPARA*}, {*MCTP1, CTSC*}, and {*PYROXD1, CYF1P2*} are functionally related. Furthermore, two differentially expressed gene pairs ({*FOXM1, PPARA*} and {*IL2R, CYFIP2*}) are involved in the vascular endothelial growth factor receptor signaling pathway and the purine ribonucleoside diphosphate metabolic process, respectively.

**Conclusion:**

This research found that the combination of computational analysis and laboratory data provided the opportunity to better characterize the relation between central colorectal cancer genes as well as possible pathways involved in the colorectal cancer.

## Introduction

 The fourth most common cancer globally is colorectal cancer (CRC), a lethal malignancy with high mortality rates. According to the National Cancer Institute (NCI), this disease is estimated to cause about 52,980 deaths in 2021. Despite major advances made in earlier years, the conventional treatment approaches, such as surgery, chemotherapy and radiation, have slight impacts on human mortality (1). Therefore, developing a comprehensive understanding of the pathways involved in CRC will effectively help to diagnosis, prevent, and treat such disease.

The progress of CRC is considered to be a stepwise procedure with the accumulation of various genetic alterations (2). Traditional approaches of detecting genetic alterations that are intricate in the development of colorectal cancer are dependent upon discovering distinct genes (3). However, analysis of single genes is insufficient to provide a proper understanding of the pathogenicity mechanisms responsible for cancer inception, development, and invasion. Research into diverse cancers through biological pathway investigation may provide a better understanding of the pathogenicity mechanisms by focusing on gene sets or pathways instead of single genes (4).

Studying gene expression patterns has come to be the backbone of recent investigations in practical genomics (5, 6). Different studies on CRC have compared the expression patterns of genes in tumor and normal tissues at different stages of the disease (7-11). In this approach, typically, the gene expression patterns of the specimens (tumor vs. adjacent normal tissues) are analyzed in the two conditions, and differentially expressed (DE) genes are determined with high statistically significant levels. Finally, a series of differentially expressed genes (DEGs) are determined for cancer detection or prognosis (12). 

Such approaches do not consider the interactions among genes, and unfortunately, the association between sets of genes and phenotype is not taken into account. Numerous dissimilar genes contribute to oncogenesis with no specific gene having a remarkably large result (13). Moreover, many studies have reported that highly correlated genes tend to be functionally correlated. This co-expression relation of genes may alternate due to the cellular state (14). Moreover, some co-expression associations may be realized in a non-healthy state, but not in a healthy state (15). 

In the current study, computational methods were used on Real-Time RT-PCR data obtained in the authors’ previous study to find the relation between central colorectal cancer genes as well as possible pathways involved in CRC. 

## Methods

**Selection of the candidate genes**

Previously, a panel of genes by Agendia, which is a classifier of robust gene expression (ColoPrint), was found to significantly improve the prognostic accuracy of pathologic factors in stage II and III CRC patients (16). Five genes (i.e. *PPARA, MCTP1*, *PYROXD1*, *IL2R, *and* CYFIP2*) were selected from this panel. Furthermore, a literature search was performed using the keywords “biomarkers,” “predict,” “diagnosis,” “colorectal cancer.” “relapse,” and “expression pattern” in the title and within the abstract. Articles discovered in the literature search were archived and filtered. Finally, a panel of genes was selected whose expression levels were reportedly changed in CRC tumors by various investigators and expected to be useful in classifying cancer staging or predicting patient risk for recurrence (Table 1).

**Collected samples**

In total, 54 CRC tissues and 48 adjacent normal tissues registered at the Rasool-e-Akram Hospital were analyzed in this study (pathological information of 3 patients was absent). The median age of patients at the time of diagnosis was 50.5 years (range = 22-79 years). Cases were composed of 26 females and 25 males. The TNM staging information of patients was as follows: 8, 14, 18, and 8 patients were at stages I, II, III, and IV, respectively. Among these samples, 26 were localized to the colon and 19 to the rectum; information for 5 cases was not available. Twenty five (48%) patients were lymph node positive (82% N1 and 18% N2) and 29 (52%) were lymph node negative.

**RNA purification and cDNA synthesis**

TriPure Isolation Reagent and RevertAid First Strand cDNA Synthesis kits were used for RNA purification (Roche Applied Sciences, Germany) and cDNA synthesis (Thermo Fisher Scientific, Germany), respectively .

**Real-time RT-PCR**

Real-Time RT-PCR using the SYBR-Green master mix was carried out by Bosch 's real-time PCR thermal cycler (Roche Applied Sciences, Germany). The amplification process was carried out in a 10 μL reaction volume using 0.1-μM vials containing 0.5 μM of each primer, 1 μL of cDNA (as template), 5 μL of SYBR-Green master mix, and 3 μL of water. The thermal cycle program was as follows: 95 °C for 5 min for the initial denaturation step, and an amplification program (95 °C for 20, 60 °C for 15, and 72 °C for 20 seconds, respectively) repeated for 40 cycles. Primers were designed with the Oligo7 software. The specificity of the primers was theoretically tested by the BLAST database. The glyceraldehyde 3-phosphate dehydrogenase (*GAPDH*) gene was selected as the housekeeping gene.

**Real-time RT-PCR raw data statistical analysis**

The real-time RT-PCR raw data for each gene was evaluated using Linreg software. Subsequently, the expression ratio results (sample group difference relative to the control group) were analyzed for significance and statistical analysis with REST and SPSS software V22.0 (SPSS, Inc., Chicago, IL). 

**Determination of functionally-related genes**

Differentially-correlated genes in disease samples compared to healthy controls were determined using the DiffCorr package to detect the functionally-related genes involved in colorectal cancer. This package can discover alteration correlations between two situations. Differentially-expressed genes with a *p*-value <0.05 were chosen as functionally-related genes.

**Gene set enrichment analysis**

To verify the functionally-related genes, gene set enrichment analysis (GSEA) was performed. GSEA is a statistical technique that tests the importance of predefined observations (17). Gene enrichment analysis was carried out on nine surveyed genes according to the gene ontology database's biological procedure. The ClueGO tool (18) (with a Kappa threshold of 0.4) was used within the Cytoscape v.3.3.0 environment (19). The right-sided hypergeometric test and the Benjamin-Hochberg correction method (20) were applied to confirm the results of the enrichment investigation. 

**Gene regulatory network construction**

Another analysis method used to verify the functionally-related genes is the gene regulatory network (GRN). A GRN is used to model regulatory mechanisms and provide a more realistic view of gene regulation. This network contains edges and nodes (genes) (regulatory associations) (21). Here, ARACNE (Algorithm for the Reconstruction of Accurate Cellular Networks) (22) was used to construct the GRN. Assembly of GRN was achieved using geWorkbench_2.6.0 software (23) applying ARACNE by considering *p*-value <0.05 as significant.

**Table 1:** Characteristic features of genes studied in this article

Name/Gene ID	Accession number	Location	Description	Biological activity
FOXM1/ 2305	NM_202002.2	12p13	Forkhead box protein M1	Transcription regulation
PPARA/ 5465	NM_001001928.2	22q13.31	Peroxisome proliferator-activated receptor alpha	Transcription regulation
PIM3/ 415116	NM_001001852.3	22q13	Serine/threonine-protein kinase pim-3	serine/threonine kinase activity
MCTP1/ 79772	NM_001297777.1	5q15	Multiple C2 domains, transmembrane 1	calcium-mediated signaling
PYROXD1/79912	NM_024854.3	12p12.1	Pyridine nucleotide-disulphide oxidoreductase domain 1	oxidoreductase
BMI1/ 648	NM_ 35226	10p12.2	Polycomb complex protein BMI-1	Transcription regulation
IL2R / 3559	NM_ 01589	10p15.1	Interleukin 2 receptor subunit alpha	Receptor/Immunity
CYFIP2 / 26999	NM_ 96F07	5q33.3	Cytoplasmic FMR1-interacting protein 2	Apoptosis, Cell adhesion
CTSC/	NM_ 53634	11q14.2	Cathepsin C	Hydrolase, Protease, Thiol protease

## Results

**Gene expression patterns in CRC patients**

 This study evaluated gene expression levels of the *FOXM1, PYROXD1, BMI1, PPARA, PIM3, IL2R, MCTP1, CYFIP2, *and *CTSC* genes in tumor and adjacent normal tissue of CRC patients. Real-time PCR data was analyzed using the REST software. Gene expression rates among cancerous and non-cancerous specimens are outlined in Table 2.

**Identification of differentially expressed genes**

A list of 9 gene pairs was obtained as the differentially expressed genes by differential correlation analysis with a *p*-value < 0.05 being considered significant. The results of this analysis are listed in Table 3.

**Gene set enrichment analysis results**

**Figure 1:**
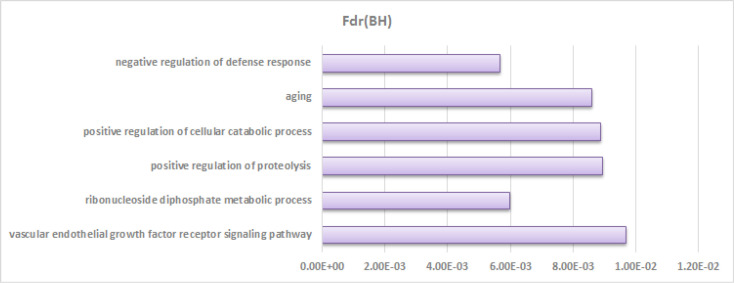
Enriched terms due to biological procedure outcome

GSEA was applied to determine biologically-relevant differentially expressed genes. The biological processes by considering FDR< 0.01 was selected. The results of such analysis are shown in Figure 1. As the terms in gene ontology, lower levels are more wide-ranging, and enriched terms lower than level 6 are not stated. The results of GSEA showed that the nine surveyed genes are stringy involved in the vascular endothelial growth factor receptor signaling pathway, purine ribonucleoside diphosphate metabolic process, positive regulation of cellular catabolic process, negative regulation of defense response, aging, and positive regulation of proteolysis. Furthermore, two differentially expressed gene pairs, i.e. {*FOXM1, PPARA*} and {*IL2R, CYFIP2*}, are involved in the vascular endothelial growth factor receptor signaling pathway and purine ribonucleoside diphosphate metabolic process, respectively (24) . The full results of enriched terms are reported in Supplementary File 1.

**Gene regulatory network results**

**Figure 2:**
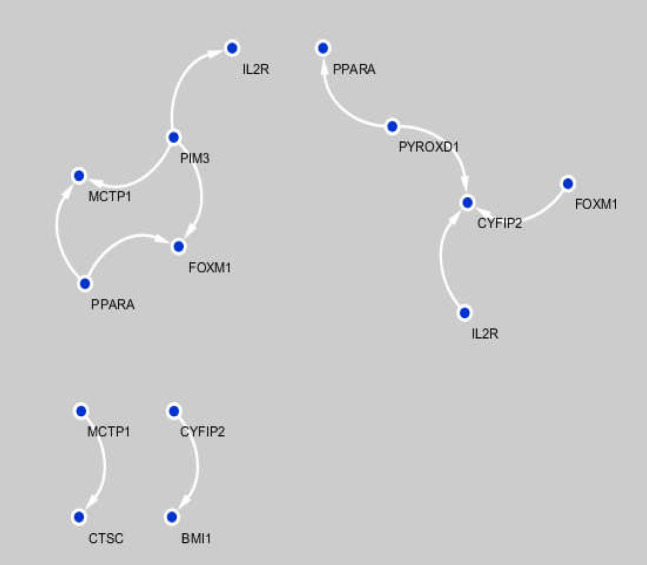
Regulatory relationships results for differentially expressed gene pairs

Functionally-related genes were evaluated using GRN. To this end, a GRN was reconstructed based on ARACNE. The information of this network is reported in Supplementary File 3. The resulting network included 9 nodes and 11 edges regarding* p*-value <0.05 as the threshold. This scheme is reported in Figure 2. The regulatory association of four differentially expressed gene pairs, i.e. {*IL2R, CYFIP2*}, {*FOXM1, PPARA*}, {*MCTP1, CTSC*}, and {*PYROXD1, CYF1P2*} can be seen without an intermediary, as was expected.

**Table 2:** Relative expression of genes investigated in this study

Gene	P-value	Changes	Rate of change
*FOXM1*	0.03	UP	2.3
*PYROXD1*	0.006	UP	6.780
*BMI1*	0.73	No significant	1.22
*PPARA*	0.7	No significant	1.076
*PIM3*	0.335	No significant	1.75
*IL2R*	0.963	No significant	0/968
*MCTP1*	0.096	No significant	2.11
*CYFIP2*	0.61	No significant	0/443
*CTSC*	0.05	UP	2.46

P value less than 0.05 (P ≤0.05) was considered statistically significant.

**Table 3:** Differential correlation analysis result

	molecule X	molecule Y	r1 (t)	p1	r2 (s)	p2	p (difference)	(r1-r2)
1	PYROXD1	BMI1	0.622313	3.05E-07	-0.25833	0.107515	3.56E-06	0.880642689
2	IL2R	CYFIP2	0.387694	0.003155	-0.24781	0.123128	0.001995546	0.635507984
3	MCTP1	PYROXD1	0.658082	3.56E-08	0.240025	0.135747	0.011015155	0.418057001
4	FOXM1	PIM3	0.535449	2.12E-05	0.064133	0.694206	0.01275803	0.471316237
5	FOXM1	PPARA	0.555447	8.85E-06	0.114893	0.48022	0.017104877	0.440554251
6	MCTP1	CTSC	0.558386	7.75E-06	0.807078	3.17E-10	0.022702373	0.248692232
7	PPARA	BMI1	0.374181	0.004498	-0.07718	0.635979	0.02804058	0.451356473
8	PYROXD1	CYFIP2	0.03347	0.806547	-0.39529	0.011585	0.035053987	0.428764472
9	CTSC	IL2R	0.386942	0.003219	-0.04247	0.794717	0.03539901	0.429409634

## Discussion

Colorectal cancer is a common malignancy that affects both men and women. It is one of the most common cancers (the third most common cancer) and the fourth leading cause of death in the world, causing an estimated 400,000 deaths each year (25).

The conventional method for CRC staging is the tumor, node, and metastasis (TNM) system. This method is based on macroscopic and microscopic morphological tests of specimens (26). Although this system has an advantage among researchers as an international language, it has many limitations as the first way to predict and prevent cancers (27). Therefore, identifying the biological pathways involved in the disease is needed to better classify colorectal cancer cases. In recent years, researchers have focused on cancer biomarkers. Recently, the introduction of gene expression profile analysis methods has led to better classification of CRC at a molecular level, indicating that colorectal cancer can be divided into different subgroups with distinct gene signatures that improves CRC diagnosis, prevention, and treatment (8, 28).

Previously, a panel of genes by Agendia, a classifier of robust gene expression (ColoPrint), was determined to significantly improve the prognostic accuracy of pathologic factors in patients with stages II and III CRC (16).

Unfortunately, in the studies on individual gene expression, the relationship between genes was not considered, and a great deal of information about the regulatory relationships between genes and biological mechanisms that is critical to the development of specific cellular conditions was not taken into account in these studies (29, 30).

This study attempted to combine computational analysis with raw laboratory data to provide the chance to find pathways associated with CRC tumorigenicity and to better characterize the colorectal cancer network.

In the current study, nine gene were selected: a) 5 genes from ColoPrint panel, selected randomly; and b) 4 other genes which are not in this panel but were cited abundantly in the literature (31-33). Then, expression levels of the selected genes were compared between CRC tissue and adjacent normal tissue. Subsequently, pathways and cross-shaped co-expression patterns (in which the expression level of two genes is directly related under one biological condition (e.g., a healthy sample), while in the other (e.g., a tumor sample) it is inversely related) were determined using computational methods (34).

In this research, the expression level of each gene was first determined using Real-Time RT PCR in tumor tissues in contrast with normal parallel tissues. Then, computational methods were used on Real-Time RT-PCR data to find the relation between central colorectal cancer genes as well as possible pathways involved in colorectal cancer.

To elucidate correlated genes between two experimental conditions (disease samples compared to healthy controls), the DiffCorr package was used (35). With this approach, gene pairs {*PYROXD1, BMI1*}, {*IL2R, CYFIP2*}, {*MCTP1, PYROXD1*}, {*FOXM1, PIM3*}, {*FOXM1, PPARA*}, {*MCTP1, CTSC*}, {*PPARA, BMI1*}, {*PYROXD1, CYFIP2*}, and {*CTSC, IL2R}* having cross-shaped co-expression patterns were identified. It seems this condition might be one of the first steps towards tumorigenicity.

The gene regulation network (GRN) models complex regulatory mechanisms to be able to check and control the gene activities, especially gene expression in a living cell. This network is represented by a directional graph made up of a set of vertices (genes) and edges (regulatory connections). These links can be activators or inhibitors. The existence of two vertices with no edges indicates that there is no regulatory relationship between them. By using such network, gene expression variation and, subsequently, gene connection alteration can be predicted during special conditions. In the current study, a GRN was reconstructed based on ARACNE. The resulting network included 9 nodes and 11 edges regarding* p*-value <0.05 as the threshold. The regulatory association of four differentially expressed gene pairs {*IL2R*, *CYFIP2*}, {*FOXM1, PPARA*}, {*MCTP1, CTSC*}, and {*PYROXD1, CYF1P2*} can be seen without any intermediary, as was expected.

Gene set enrichment analysis showed that genes with a high correlation tend to be functionally connected and usually involved in the same biological pathway (29). Therefore, gene set enrichment analysis was used to provide a comprehensive understanding of the pathways involved in this disease. GSEA results showed that the nine surveyed genes are strongly involved in the vascular endothelial growth factor receptor signaling pathway, purine ribonucleoside diphosphate metabolic process, positive regulation of cellular catabolic process, negative regulation of defense response, aging, and positive regulation of proteolysis. Furthermore, two differentially expressed gene pairs ({*FOXM1, PPARA*} and {*IL2R, CYFIP2*}) are jointly involved in the vascular endothelial growth factor receptor signaling pathway and purine ribonucleoside diphosphate metabolic process, respectively.

Based on GRN results, the functional relationship between two genes pairs ({*IL2R, CYFIP2*} and {*FOXM1, PPARA*}) in colorectal cancer was found to be statistically significant by the differential co-expression method; this result was also confirmed by GRN and GSEA methods. Furthermore, he two biological processes of vascular endothelial growth factor signaling pathway and metabolic diphosphate ribonucleotide process are considered as important biological processes which play central roles in colorectal cancer.

As expected, functionally related genes were involved in the same biological procedure. The two gene pairs {*IL2R, CYFIP2*} and {*FOXM1, PPARA*} are involved in the vascular endothelial growth factor signaling pathway and ribonucleotide diphosphate metabolic process, respectively. In the following, each pathway is discussed separately.

**Vascular endothelial growth factor signaling pathway**

The fact that tumor growth may be developed by extended new blood vessel formation was stated more than a century ago (36). Angiogenesis is a widely multifarious and multistep procedure that increases vascularity (37). One of the more critical regulators of the angiogenesis process is the vascular endothelial growth factor (VEGF) and its receptors (VEGFRs). The vascular endothelial growth factor (VEGF) family are soluble protein growth factors that regulate multiple endothelial cell functions, including mitogenesis (38). Because blood vessels provide oxygen and other essential nutrients for cancer cells, they are essential for the progression and metastasis of solid tumors (39). The fundamental role of VEGF in stimulating tumor angiogenesis and the pathogenesis of different cancers has led to the improvement of agents that aim this pathway as a significant new treatment in cancer therapy (40). In this research, it was determined that differentially expressed gene pairs {*FOXM1, PPARA*} are involved in the vascular endothelial growth factor signaling pathway. 

The Forkhead box protein M1 transcription factor* (FOXM1)* has been shown to have a crucial function in the cell cycle progress. Recently, a growing number of studies have described *FOXM1* as a key oncogenic transcription factor, as it can promote tumor progression (31). 

In addition, previous studies have shown that *FOXM1* is involved in the development of blood vessels for normal embryonic and fetal tissue (41, 42). Furthermore, several studies have displayed that *FOXM1* regulates the expression of genes involved in the vascular endothelial growth factor signaling pathway and promotes angiogenesis in various cancers as well as pulmonary arterial hypertension (42, 43). *PPARA *is a nuclear protein which belongs to the subfamily of peroxisome proliferator-activated receptors. *PPARA* was in the gene panel of ColoPrint with overexpression; nevertheless neither this article nor any other article has shown how this gene is involved in the development of colorectal cancer (44). Recently, Berger *et al.* conducted a study similar to the current research in which they analyzed the gene networks and pathways involved in the growth of cancer cells (45). In their study, they showed indirectly that a gene panel expression including *FOXM1* and *PPARA* along with other genes plays a role in creating a healthy liver. Furthermore, alterations in this panel gene expression pattern are involved in causing various liver diseases.

However, the current study has shown for the first time that differentially expressed gene pairs {*FOXM1, PPARA*} and {*IL2R, CYFIP2*} are involved in the vascular endothelial growth factor signaling pathway. 

**Ribonucleoside diphosphate metabolic process**

The second pathway is the ribonucleoside diphosphate metabolic process. In biochemistry, ribonucleotides are considered as the basic building blocks of RNA. They are also involved in other cellular functions such as cell regulation, cell signaling, and the energy currency in organisms through converting ribonucleotides to adenosine triphosphate (ATP) (46). The current study reports for the first time that differentially expressed gene pairs {*FOXM1, PPARA*} and {*IL2R, CYFIP2*} are involved in the ribonucleotide diphosphate metabolic process.

This study determined that gene pairs {*PYROXD1, BMI1*}, {*IL2R, CYFIP2*}, {*MCTP1, PYROXD1*}, {*FOXM1, PIM3*}, {*FOXM1, PPARA*}, {*MCTP1, CTSC*}, {*PPARA, BMI1*}, {*PYROXD1, CYFIP2*}, {*CTSC, IL2R*} have cross-shaped co-expression patterns. Furthermore, it was determined that the two gene pairs {*IL2R, CYFIP2*} and {*FOXM1, PPARA*} are involved in the vascular endothelial growth factor signaling pathway and the ribonucleotide diphosphate metabolic process, respectively. These two pathways are considered as important biological processes in colorectal cancer. This research displays the significance of focusing on gene relations rather than single genes for understanding the main biological processes and network pathways involved in colorectal cancer.
